# Determinants of right heart size and function in repaired Tetralogy of Fallot

**DOI:** 10.1186/1532-429X-16-S1-P127

**Published:** 2014-01-16

**Authors:** Michael A Quail, Ming Chern Leong, Oliver Tann, Marina Hughes, Vivek Muthurangu, Andrew Taylor

**Affiliations:** 1 Centre for Cardiovascular Imaging, Institute of Cardiovascular Science, London, UK; 2 Great Ormond Street Hospital for Children, London, UK

## Background

Tetralogy of Fallot (ToF) is associated with late right ventricular (RV) dilatation and dysfunction. However the relative contribution of the various haemodynamic lesions on RV growth have not been precisely defined, due to small sample sizes in prior studies. In this study, we investigated the independent determinants of RV size and function in a large single centre population.

## Methods

417 patients (56% male) with repaired ToF who underwent cardiovascular magnetic resonance (CMR) imaging between 2003 and 2012 were included. Median age at CMR was 18yrs (IQR 12-29yrs). Data from these patients were used create multivariate linear regression models of RV end diastolic volume (EDV), end systolic volume (ESV), and ejection fraction (EF).

## Results

RV EDV was positively associated with pulmonary regurgitant (PR) volume (β 0.59, p < 1e^-6^), tricuspid regurgitant (TR) volume (β 0.24, p < 1e^-6^), body surface area (β 0.08, p < 1e^-6^), and age (β 0.20, p < 1e^-6^). Significant residual outflow tract stenosis (β -0.10, p = 0.01), branch pulmonary artery stenosis (β -0.09, p = 0.02) and RV EF (β -0.38, p < 1e^-6^) were negatively associated with RV EDV (Model, R = 0.81). Initial palliation with a Blalock-Taussig Shunt (β -0.234, p = 0.0001) was associated with lower RV EF. TR volume was associated with higher RV EF (β -0.13, p = 0.03), (Model, R = 0.26); residual outflow tract obstruction was not associated with RV EF. RV ESV had similar associations to RV EDV, except residual outflow tract obstruction and branch PA stenosis were not associated, and RVEF was the most influential covariate (Model, R = 0.83).

## Conclusions

In this large, adequately powered study, we report a comprehensive model of RV size and function in patients with repaired ToF. Unsurprisingly PR, TR and EF, are the most important covariates associated with RV EDV. However, we have also demonstrated the contribution of novel independent covariates, such as residual outflow tract and branch pulmonary artery stenosis on reduced RV EDV, which could only be identified due to sufficient statistical power. We also report for the first time an association of prior BT shunt with reduced RV function. These data provide important clinical insights, suggesting that strategies which avoid complete abolition of pulmonary stenosis could produce long-term clinical benefit.

## Funding

British Heart Foundation.

